# Activation of TRPV1 by capsaicin induces functional Kinin B_1 _receptor in rat spinal cord microglia

**DOI:** 10.1186/1742-2094-9-16

**Published:** 2012-01-20

**Authors:** Sébastien Talbot, Jenny Pena Dias, Karim Lahjouji, Maurício Reis Bogo, Maria Martha Campos, Pierrette Gaudreau, Réjean Couture

**Affiliations:** 1 Department of Physiology, Faculty of Medicine, Université de Montréal, C.P. 6128, Succursale Centre-ville, Montréal, H3C 3J7, Québec, Canada; 2 Department of Molecular Biology, Faculty of Biosciences, Pontifícia Universidade Católica do Rio Grande do Sul, Porto Alegre, 90619-900, Rio Grande do Sul, Brasil; 3 National Institute for Translational Medicine (INCT-TM), Porto Alegre, Rio Grande do Sul, Brasil; 4 Institute of Toxicology and Pharmacology, Pontifícia Universidade Católica do Rio Grande do Sul, Porto Alegre, 90619-900, Rio Grande do Sul, Brasil; 5 Faculty of Dentistry, Pontifícia Universidade Católica do Rio Grande do Sul, Porto Alegre, 90619-900, Rio Grande do Sul, Brasil; 6 Laboratory of Neuroendocrinology of Aging, Université de Montréal, CHUM Research Center, Angus Technopole, Montréal, H1W 4A4, Québec, Canada

**Keywords:** Bradykinin, B_1 _receptors, TRPV1, capsaicin, oxidative stress, thermal hyperalgesia

## Abstract

**Background:**

The kinin B_1 _receptor (B_1_R) is upregulated by pro-inflammatory cytokines and oxydative stress, which are enhanced by transient receptor potential vanilloid subtype 1 (TRPV1) activation. To examine the link between TRPV1 and B_1_R in inflammatory pain, this study aimed to determine the ability of TRPV1 to regulate microglial B_1_R expression in the spinal cord dorsal horn, and the underlying mechanism.

**Methods:**

B_1_R expression (mRNA, protein and binding sites) was measured in cervical, thoracic and lumbar spinal cord in response to TRPV1 activation by systemic capsaicin (1-50 mg/kg, s.c) in rats pre-treated with TRPV1 antagonists (capsazepine or SB-366791), the antioxidant N-acetyl-L-cysteine (NAC), or vehicle. B_1_R function was assessed using a tail-flick test after intrathecal (i.t.) injection of a selective B_1_R agonist (des-Arg^9^-BK), and its microglial localization was investigated by confocal microscopy with the selective fluorescent B_1_R agonist, [N^α^-bodipy]-des-Arg^9^-BK. The effect of i.t. capsaicin (1 μg/site) was also investigated.

**Results:**

Capsaicin (10 to 50 mg/kg, s.c.) enhanced time-dependently (0-24h) B_1_R mRNA levels in the lumbar spinal cord; this effect was prevented by capsazepine (10 mg/kg, i.p.; 10 μg/site, i.t.) and SB-366791 (1 mg/kg, i.p.; 30 μg/site, i.t.). Increases of B_1_R mRNA were correlated with IL-1β mRNA levels, and they were significantly less in cervical and thoracic spinal cord. Intrathecal capsaicin (1 μg/site) also enhanced B_1_R mRNA in lumbar spinal cord. NAC (1 g/kg/d × 7 days) prevented B_1_R up-regulation, superoxide anion production and NF-kB activation induced by capsaicin (15 mg/kg). Des-Arg^9^-BK (9.6 nmol/site, i.t.) decreased by 25-30% the nociceptive threshold at 1 min post-injection in capsaicin-treated rats (10-50 mg/kg) while it was without effect in control rats. Des-Arg^9^-BK-induced thermal hyperalgesia was blocked by capsazepine, SB-366791 and by antagonists/inhibitors of B_1_R (SSR240612, 10 mg/kg, p.o.), glutamate NMDA receptor (DL-AP5, 10 μg/site, i.t.), substance P NK-1 receptor (RP-67580, 10 μg/site, i.t.) and nitric oxide synthase (L-NNA, 10 μg/site, i.t.). The B_1_R fluorescent agonist was co-localized with an immunomarker of microglia (Iba-1) in spinal cord dorsal horn of capsaicin-treated rats.

**Conclusion:**

This study highlights a new mechanism for B_1_R induction via TRPV1 activation and establishes a link between these two pro-nociceptive receptors in inflammatory pain.

## Background

Kinins are neuroactive peptides involved in pain and inflammation [1-4]. They act through the activation of two G-protein-coupled receptors (GPCR) denoted as B_1 _(B_1_R) and B_2 _(B_2_R) [5,6]. The B_2_R, activated by bradykinin (BK) and Lys-BK, is widely and constitutively expressed in central and peripheral tissues. The BK metabolites, des-Arg^9^-BK and Lys-des-Arg^10^-BK, are the preferential agonists of B_1_R. Whereas the B_1_R is virtually absent in healthy conditions, it is upregulated after exposure to pro-inflammatory cytokines, bacterial endotoxins and hyperglycaemia-induced oxidative stress [7-10]. The induction of B_1_R involves the transcriptional nuclear factor NF-κB and MAP-kinase/P38 pathways [6,11]. We have reported that spinal injection of B_1_R agonist causes transient thermal hyperalgesia in type 1 diabetic rats due to release of sensory pro-inflammatory mediators, notably substance P (SP), prostaglandins and nitric oxide [1]. Furthermore, B_1_R antagonists reverse thermal hyperalgesia and allodynia in various models of type 1 and type 2 diabetes [4,12-15].

The transient receptor potential vanilloid subtype 1 (TRPV1) is known as a non-selective cationic channel expressed in primary sensory C-fibers [16] and microglia [17]. Its activation increases both calcium and sodium influx [16]. TRPV1 knockout mice do not display thermal hyperalgesia[18-20]. TRPV1 can be sensitized through the phosphorylation of its C-terminal end by protein kinases A and/or C [21,22]. It is activated by a variety of stimuli such as heat > 43°C [16], acidification [23], BK [24], nerve growth factor [24] and oxidative stress [25]. It was recently shown that TRPV1 activation by capsaicin increases reactive oxygen species (ROS) production in mouse dorsal root ganglion (DRG) neurons [26]. TRPV1-induced ROS production is thought to involve increased cytosolic calcium influx and activation of NADPH oxidase [27]. Moreover, it has been suggested that selective TRPV1 inhibition reduces the pro-oxidant capacity of microglial NADPH oxidase [28].

This study was undertaken to determine whether TRPV1 activation by capsaicin could enhance expression of the pro-nociceptive B_1_R since both receptors are involved in thermal hyperalgesia. Moreover, microglial TRPV1 activation enhances pro-inflammatory cytokines and oxidative stress, both known to trigger B_1_R induction through the NF-κB pathway. Thus, microglia can be considered to be a strategic target for B_1_R expression as evidenced in a diabetic model of pain neuropathy [29,30]. Our main objectives were to determine: 1- the role of oxidative stress and pro-inflammatory cytokines in capsaicin-induced B_1_R upregulation; 2- whether newly induced B_1_R is functional and could induce thermal hyperalgesia through release of spinal cord mediators; and 3- the presence of B_1_R on microglia in the spinal dorsal horn of capsaicin-treated rats by confocal microscopy.

## Methods

### Experimental animals and care

All research procedures and the care of the animals were in compliance with the guidelines of the Committee for Research and Ethical Issues of IASP and were approved by the Animal Care Committees of Université de Montréal and Pontificia Universidade Católica do Rio Grande do Sul. Male Sprague-Dawley rats (200-225 g; Charles River, St-Constant, Qc, Canada and CEMIB, UNICAMP, Brasil) were housed two per cage, under controlled conditions of temperature (23°C) and humidity (50%), on a 12 h light-dark cycle (until surgery) and allowed free access to normal chow diet (Charles River Rodent) and tap water.

### Intrathecal implantation of catheter and capsaicin treatment

Four days after arrival, rats were anaesthetized with isoflurane and chronically implanted with an indwelling intrathecal (i.t.) polyethylene catheter (PE-10; Intramedic, Clay Adams, NJ, USA) at the vertebral lumbar level (L3 to L6) through an incision made in the dura at the atlanto-occipital junction [31]. The canula was secured to the skull through two small parallel segments of PE-60 glued with cyano-acrylate. Rats with apparent abnormal behaviour or motor deficits were euthanized with CO_2 _inhalation. Rats were housed permanently in the testing laboratory under continuous light to prevent the release of endogenous opioids which could alter nociceptive threshold [32]. One week after surgery, six groups of 7-8 rats received a single dose of capsaicin (1, 5, 10, 15, 25 and 50 mg/kg), injected subcutaneously at the lumbar back skin level under mild anaesthesia with pentobarbital (45 mg/kg, i.p.). The highest dose of 50 mg/kg was injected in two doses of 25 mg/kg at 12 h intervals. Rats were used 24 h after capsaicin injection, except for rats used for time-course experiments (treated for: 0, 8, 24 or 48 h). To evaluate directly the capacity of spinally expressed TRPV1 to induce B_1_R expression, one group of rats received 1 μg of capsaicin intrathecally at the lumbar region of the spinal cord. Control rats received vehicle only (10% ethanol, 10% Tween-80 and 80% saline 0.9%). Responses to des-Arg^9^-BK were measured using a tail-flick test prior to and 24 h after capsaicin injection.

### Tail-flick test protocol

Experiments were always started in the morning at around 10:00 H AM. Awake rats were placed in a plastic restraining box. The nociceptive threshold was taken as the reaction-time to remove the tail from a source of noxious heat stimulus using either a tungsten lamp or a hot water bath (50 ± 1°C) [33]. The average tail-flick reaction times of control rats were 9.4 ± 0.2 s and 5.8 ± 0.3 s, when assessed with the tungsten lamp and the hot water bath as heat stimuli, respectively. A 25 s cut-off time was used to prevent tissue damage [31]. All rats were tested for a maximum of four consecutive days. During the first two days, rats received 10 μl of artificial cerebrospinal fluid (aCSF) as training experiments and to ensure that the intrathecal catheter was patent. On the subsequent days, each testing trial lasted 45 min and consisted of 9 measurements of tail-flick latency, spaced by 5 min intervals. The initial three measurements were used to determine baseline latency [31]. One minute prior to the 4^th ^reading (t = 15 min), the vehicle (aCSF) or an inhibitor was i.t. injected. Twelve min later, the tested agonist (des-Arg^9^-BK) or substance P (SP) (as positive control) was administered through the same route (t = 26 min), and its effect on the tail-flick latency was measured 1 min later (t = 27 min). Three subsequent readings were made to assess the effect of the agonist on the nociceptive threshold. The study design for time and agonist doses (des-Arg^9^-BK, 9.6 nmol; SP, 6.6 nmol) was based on previous studies showing that SP and des-Arg^9^-BK evoke a transient 5-10 min thermal hyperalgesia that peaks 1 min post-injection [1,31,34]. Drugs and tested agonists were intrathecally injected using a 50 μl Hamilton syringe with a total volume of 10 μl. The catheter, with a void volume of 10 μl, was immediately flushed after drug injection by the administration of 15 μl aCSF.

### Pharmacological treatments

The hyperalgesic response induced by SP and des-Arg^9^-BK in the tail-flick test was characterized by determining the effects of the following **s**elective antagonists/inhibitors (10 μg/site) administered intrathecally 15 min prior to the agonist: nitric oxide synthase (NOS): L-N^G^-Nitroarginine (L-NNA) [35], neurokinin-1 (NK-1) receptor: [Imiro-1 (methoxy-2 phenyl)-2ethyl]-2 diphenyl-7.7 perhydroisoindolone-4-(3aR, 7aR) (RP-67580) [36], and N-methyl-D-aspartic acid (NMDA) receptor: D, L-2-amino-5- phosphonovaleric acid (DL-AP5) [37]. SSR240612 was used in this study as a selective and orally active B_1_R antagonist. Dosage of SSR240612 (10 mg/kg, 3 h pre-treatment) was based on previous studies showing that: i) des-Arg^9^-BK-induced paw oedema in mice is inhibited by 1 h pre-treatment with 3 and 10 mg/kg, p.o. of SSR240612 [38], and ii) allodynia and high systolic blood pressure induced by insulin resistance are prevented by a 3 h pre-treatment with SSR240612 (IC_50 _of 5.5-7.1 mg/kg) [4,39]. Finally, we used two generations of TRPV1 antagonists (capsazepine and SB-366791) [16] to unequivocally demonstrate that capsaicin effects are attributable to TRPV1 stimulation. Capsazepine was administered intraperitoneally 1 h before capsaicin challenge at a dose of 10 mg/kg [40] or intrathecally at a dose of 10 μg/site [41]. SB-366791 [N-(3-methoxyphenyl)-4-chlorocinnamide] is known as a more selective and more potent TRPV1 antagonist than capsazepine [42]. SB-366791 was administered intraperitoneally 1 h before capsaicin challenge at a dose of 1 mg/kg [43] or intrathecally at a dose of 30 μg/site [44].

### Antioxidant treatment

Rats without intrathecal catheter received every day for a period of 1-week the potent antioxidant N-acetyl-L-cysteine (NAC) (1 g/kg/d) or the vehicle (sterile water) by gavage [8]. On the 6^th ^day of treatment with NAC, rats received a single injection of capsaicin (15 mg/kg, s.c.) or its vehicle. Rats were sacrificed 24 h later under inhalation with CO_2_.

### Tissue preparation for autoradiography and microscopy

Twenty-four hours after capsaicin treatment, rats were anaesthetised with CO_2 _inhalation and then decapitated. Lower-lumbar (L3-L6) spinal cord was removed and frozen in 2-methylbutane (cooled at -55 ± 5°C with liquid nitrogen) and stored at -80°C. Spinal cords were mounted in a gelatin block and serially cut into 20-μm thick coronal sections with a cryostat. The sections were thaw-mounted on 0.2% gelatin-0.033% chromium potassium sulfate-coated slides and kept at -80°C for 1 month to allow sections adhesion to the coverslip glasses.

### Quantitative autoradiography

The density of kinin B_1_R binding sites was measured with the radioligand [^125^I]-HPP-desArg^10^-Hoe140 (3-(4 hydroxyphenyl) propionyl-desArg^9^-D-Arg^0^[Hyp^3^, Thi^5^, D-Tic^7^, Oic^8^]bradykinin) used at a concentration of 200 pM (specific activity: 2000 cpm/fmol) as described previously [3,8,15,29]. Non-specific binding was determined for each capsaicin doses in the presence (1 μM) of the selective B_1_R antagonist R-715 (AcLys[D-βNal^7^, Ile^8^]des-Arg^9^-BK) [45]. Scientific Imaging Films BIOMAX ™ MR^® ^(Amersham Pharmacia Biotech Canada) were juxtaposed onto the slides in the presence of [^125^I]-microscales and exposed at room temperature for 7 days. Autoradiograms were quantified by densitometry using an MCID™ image analysis system (Imaging Research, St. Catharines, ON, Canada). Briefly, twenty measurements were made throughout the spinal cord dorsal horn in 3 to 8 rats per group. A standard curve from [^125^I]-microscales was used to convert density levels into fentomoles per milligram of protein [46]. Specific binding was determined by subtracting values of nonspecific binding from that of total binding.

### Confocal microscopy

The exhaustive protocol and specificity of the fluorescent B_1_R agonist have been described elsewhere [29]. Sections were dissected out under a binocular microscope to specifically isolate the spinal cord dorsal horn. Briefly, unfixed 20-μm sections were exposed for 90 min to 50 μM [N^α^-bodipy]-des-Arg^9^-BK (N^α^-4,4,-difluoro-5,7-dimethyl-4-bora-3a,4a-diaza-s-indacene-3-propionic acid succinimidyl ester-des(Arg^9^)-bradykinin) to label the B_1_R. Slices were then incubated with a blocking buffer (25 mM PIPES buffer supplemented with 3% bovine serum albumin (BSA) and 3% donkey serum) to prevent non-specific labeling. Antibody was diluted in blocking buffer. Rabbit anti-ionized calcium binding adapter molecule 1 (anti-Iba-1, Wako, Richmond, VA, Cat No: #019-19741) at a concentration of 2 μg/ml was used to label microglia [30]. The secondary antibody was rhodamine anti-rabbit 1:500 (Chemicon, Hornby, ON, Cat No: # R-6394).

### Measurement of superoxide anion

*In situ *levels of O_2 _in the spinal cord were evaluated with the oxidative fluorescent dye dihydroethidium (DHE) as described earlier [47]. Cells are permeable to DHE which specifically react with O_2 _to produce ethidium bromide (EtBr) which is trapped by intercalation with DNA. EtBr is excited at 518 nm with an emission spectrum of 605 nm. Twenty-four hours post-capsaicin, rats were anaesthetized with CO_2 _inhalation and then decapitated. The lumbar spinal cord (L3-L6) was isolated and serially cut into 20-μm thick sections and placed on glass slides. On the day of experiment, slides were exposed for 30 min in a light-protected humidified chamber at 37°C to DHE (2 μM) for superoxide anion labeling and to TO-PRO-3 1:5000 (Molecular Probes, Eugene, OR, USA) for DNA staining. Sections were then washed 3 times in sterile phosphate buffered saline (PBS, pH7.4), coverslipped and observed using a confocal microscope (Leica Microsystem Co., Germany). Slides from the four groups (control, control + NAC, capsaicin-treated, capsaicin-treated + NAC) were processed and imaged in parallel. Laser settings were identical for acquisition of images from all sections. Computer based analysis was performed with Image J software and calculated using the following equation: I=∑ I/(A/N), where I is the fluorescence intensity, ∑ I is the summation of all nuclei intensity, A is the total area of the nuclei, and N is the number of nuclei used [47]. Data are expressed as an average of total nuclei fluorescence quantified on at least 4 nuclei/section on 4 sections/rat from 4 different rats. Positive pixels regarding the co-localisation of B_1_R and Iba-1 were determined following the subtraction of mean pixel background level intensity from total pixels.

### SYBR green-based quantitative real-time PCR

Twenty-four hours after capsaicin injection, rats were anaesthetized with CO_2 _inhalation and then decapitated. Cervical (C2-C4), thoracic (T3-T5) and lumbar spinal cord (L3-L6) segments were isolated and approximately 10 mg of tissue were put in RNA*later *stabilization reagent (QIAGEN, Valencia, CA, USA). The protocol for mRNA extraction, cDNA generation, SYBR green-based quantitative RT-PCR and quantification has been described elsewhere [29]. The PCR conditions were as follows: 95°C for 15 min, followed by 46 cycles at 94°C for 15 s, 60°C for 30 s and 72°C for 30 s. Real-time PCR primer pairs were designed using Vector NTI and are presented in Table 1.

**Table 1:** Real-time PCR primer pairs.

	Sequences			Position			Gen Bank
18 S forward	5'	TCA ACT TTC GAT GGT AGT CGC CGT	3'	363	-	386	X01117
18 S reverse	5'	TCC TTG GAT GTG GTA GCC GTT TCT	3'	470	-	447	

B_1 _receptor forward	5'	GCA GCG CTT AAC CAT AGC GGA AAT	3'	367	-	391	NM_030851
B_1 _receptor reverse	5'	CCA GTT GAA ACG GTT CCC GAT GTT	3'	478	-	454	

IL-1β forward	5'	TGT CAC TCA TTG TGG CTG TGG AGA	3'	247	-	270	NM_031512
IL-1β reverse	5'	TGG GAA CAT CAC ACA CTA GCA GGT	3'	411	-	388	

TNF-α forward	5'	ATG ATC CGA GAT GTG GAA CTG GCA	3'	160	-	183	NM_012675
TNF-α reverse	5'	AAT GAG AAG AGG CTG AGG CAC AGA	3'	257	-	234	

### Western blot

The extensive procedure has been described elsewhere [48]. Briefly, 24 h after capsaicin treatment, rats were anaesthetized with CO_2 _inhalation and then decapitated. The lumbar spinal cord (L3-L6) was isolated and approximately 50 mg of tissue were put in PBS containing a cocktail of proteases inhibitors (Sigma-Aldrich, Canada). Antibodies against B_1_R, p65NF-kB and dynein (internal control) are presented in Table 2.

**Table 2:** Western Blot primary and secondary antibodies.

Primary antibodies		Molecular weight	Dilution	Source	
rabbit polyclonal anti-rat B_1_R		37 kDa	1:1000	Réjean Couture's Laboratory [48]	
rabbit polyclonal anti-rat p65NF-κB		65 kDa	1:1000	SantaCruz Biotechnology (Cat number Sc-8008)	
mouse monoclonal anti-rat dynein		70 kDa	1:20000	SantaCruz Biotechnology (Cat umber Sc-13524)	

**Secondary antibodies**		**Protein detected**	**Dilution**	**Source**	

HRP-linked goat anti-rabbit		B_1_R, p65NF-κB	1:5000	SantaCruz Biotechnology (Cat number Sc-2077)	
HRP-linked goat anti-mouse		dynein	1:5000	SantaCruz Biotechnology (Cat number Sc-2005)	

### Drugs and solutions

Des-Arg^9^-BK and SP were purchased from Bachem Bioscience Inc. (King of Prussia, PA, USA). RP-67580 and SB-366791 were purchased from Tocris Cookson Inc (Ellisville, MO, USA). The B_1_R antagonist, SSR240612 [(2R)-2-[((3R)-3-(1,3-benzodioxol-5-yl)-3-[[(6-methoxy-2-naphthyl)sulfonyl]amino]propanoyl)amino]-3-(4-[[2R,6S)-2,6-dimethylpiperidinyl]methyl]phenyl)-N-isopropyl-N-methylpropanamide,fumarate], was kindly provided by Sanofi-Aventis (Montpellier, France). HPP-des-Arg^10^-Hoe140 was synthesized at the Research Institute of Biotechnology, National Research Council of Canada (Montreal, Qc, Canada). R-715 was kindly provided by Dr Fernand Gobeil (Pharmacology, University of Sherbrooke, Sherbrooke, Qc, Canada). [N^α^-bodipy]-des-Arg^9^-BK was synthesized by Dr. Pierrette Gaudreau (Research Center CHUM, Université de Montréal, Montréal, Qc, Canada) [29]. Dihydroethidium was obtained from Molecular Probes (Invitrogen Corporation, Carisbad, CA, USA) and suspended in DMSO at a concentration of 10^-3 ^M, and stored at -20°C until use. Capsaicin, capsazepine, NAC, DL-AP5 and L-NNA were purchased from Sigma-Aldrich Canada, Ltd (Oakville, ON, Canada). For i.t. injections, SP, des-Arg^9^-BK, DL-AP5, L-NNA, RP-67580, SB-366791 and capsazepine were dissolved in aCSF while SB-366791 and capsazepine were dissolved in sterile saline for i.p. injections. SSR240612 was dissolved in dimethyl sulfoxide (DMSO, 0.5% v/v), ethanol (5% v/v) and Tween-80 (5% v/v). The solution was completed in distilled water. The drug was administered orally by gavage in a volume of 1 ml per 100 g of body weight [39]. Capsaicin was solubilized in a mixture of 10% ethanol, 10% Tween-80 and 80% saline 0.9%.

### Statistical analysis

Data were expressed as means ± S.E.M. of values obtained from *n *rats. Only one treatment was given to a rat. For the tail-flick test, data were calculated as a percentage of the maximum possible effect (% MPE) according to the following formula: % MPE = 100 × (drug latency minus baseline latency/cut-off time minus baseline latency) [1]. Statistical significance was determined with Student's *t*-test for paired samples or with one-way analysis of variance (ANOVA) followed by the Bonferroni post-test for multiple comparisons. Data for DHE labelling intensity were analysed with the non-parametric Kruskal-Wallis post-test. Probability (P) values less than 0.05 were considered to be statistically significant.

## Results

### Increased B_1_R expression by capsaicin-induced TRPV1 activation

**Figure 1:**
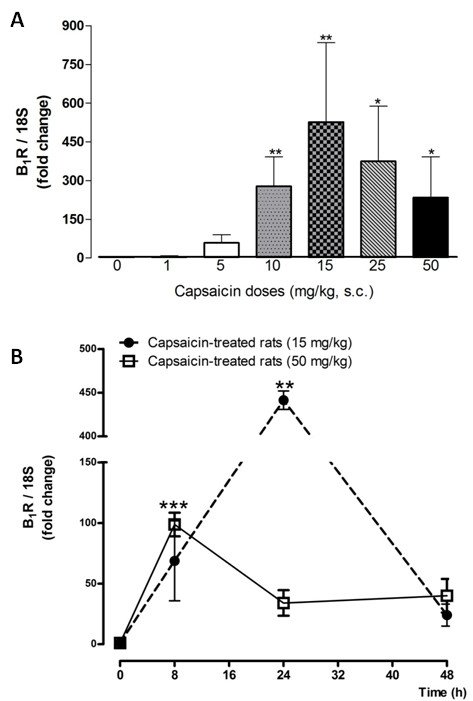
**(A) Changes of B_1_R mRNA levels in the lumbar spinal cord of capsaicin-treated rats (0-50 mg/kg, s.c.)**. Data represent the means ± S.E.M. of 4 to 7 rats per group (a single dose of capsaicin per group, 24 h earlier). Comparison with the 0 mg/kg group is indicated by * P < 0.05; ** P < 0.01. (B) Time-course effect (0 - 48 h) of 15 mg/kg (•) and 50 mg/kg (□) capsaicin on lumbar spinal cord B_1_R mRNA expression levels. Data represent the means ± S.E.M. of 4 rats per group. Comparison with the 0 h group (*) is indicated by ** P < 0.01; *** P < 0.001. Shown are the responses in rats housed under constant light (A) or a 12 h-12 h light-dark cycle (B).

**Figure 2:**
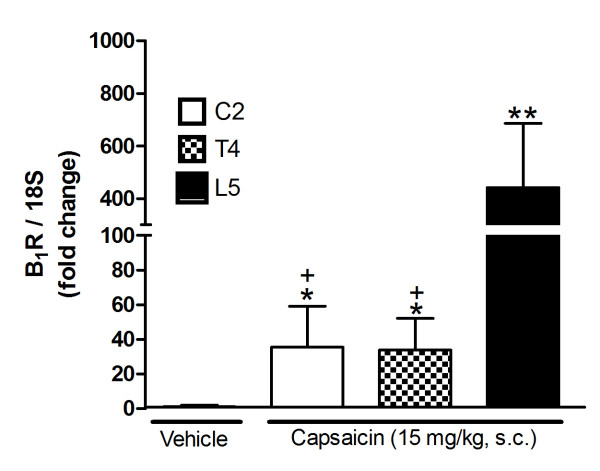
**Effect of 24 h capsaicin (15 mg/kg, s.c.) or its vehicle on B_1_R mRNA expression levels in cervical (C2), thoracic (T4) and lumbar (L5) segments of rat spinal cord**. Data represent the means ± S.E.M. of 4 rats per group. Comparison with vehicle (*) or lumbar segment (+) is indicated by +,* P < 0.05; ** P < 0.01.

**Figure 3:**
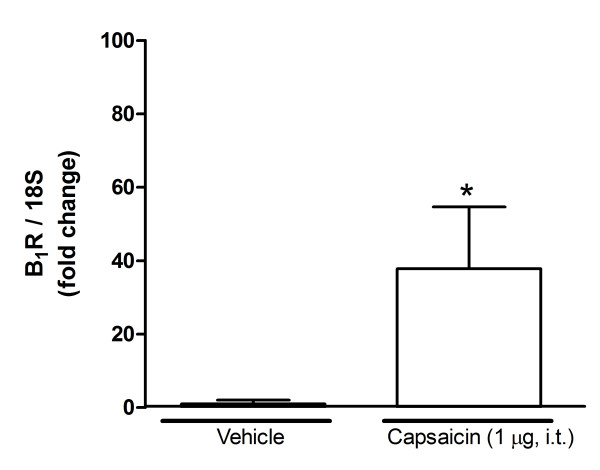
**Effect of intrathecally injected capsaicin (1 μg/site, 24 h) or its vehicle on B_1_R mRNA expression level in lumbar spinal cord**. Data represent the means ± S.E.M. of 4 rats per group. Comparison with vehicle (*) is indicated by * P < 0.05.

A 24-h-capsaicin treatment (10-50 mg/kg, s.c.) increased significantly B_1_R mRNA levels in rat lumbar spinal cord when compared with vehicle-treated rats. A dose of 15 mg/kg caused the highest increase of gene expression (500-fold) at 24 h and the effect was almost gone at 48 h (Figure 1). In comparison, a dose of 50- mg/kg capsaicin caused a smaller enhancing effect on B_1_R mRNA, which peaked at 8 h and declined thereafter (Figure 1B). The increasing effect of 15 mg/kg capsaicin on B_1_R mRNA was significantly less at the thoracic (T4) and cervical (C2) spinal cord levels (Figure 2). Intrathecal capsaicin (1 μg/site) reproduced to some extent the effects of systemic capsaicin, as it significantly increased B_1_R mRNA in the lumbar spinal cord in comparison with vehicle-treated rats 24 h post-treatment (Figure 3).

**Figure 4:**
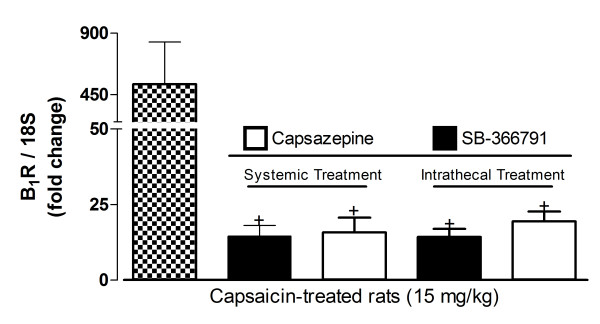
**Effect of 1 h pre-treatment with TRPV1 antagonists (capsazepine (10 mg/kg, i.p. or 10 μg/site, i.t.) and SB-366791(1 mg/kg, i.p. or 30 μg/site, i.t.)) or vehicle (filled dotted bars) on the increased lumbar spinal cord B_1_R mRNA level induced by capsaicin (15 mg/kg, s.c.)**. Data represent the means ± S.E.M. of 4 rats per group. Comparison with capsaicin pre-treated with the vehicle is indicated by + P < 0.01.

Capsaicin-induced B_1_R up-regulation is likely associated with TRPV1 activation as the increased B_1_R mRNA expression evoked by 15 mg/kg capsaicin was prevented by capsazepine (10 mg/kg, i.p. or 10 μg/site, i.t.) and SB-366791 (1 mg/kg, i.p. or 30 μg/site, i.t.), when injected 1 h beforehand (Figure 4).

**Figure 5:**
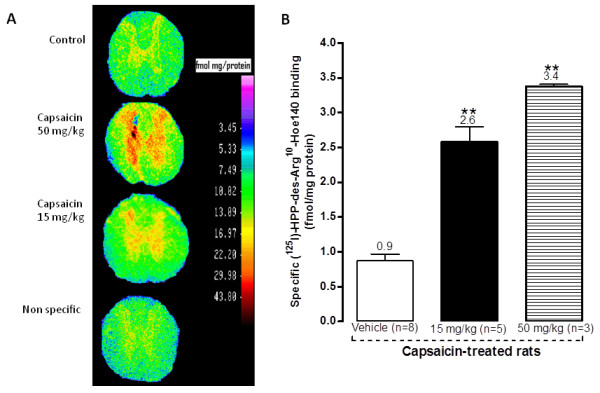
**Autoradiograms (A) and quantitative densitometric analysis (B) of specific B_1_R binding sites in the grey matter of lumbar spinal cord of rats treated with capsaicin (15 and 50 mg/kg, s.c.) or vehicle, 24 h earlier. Data represent the means ± S.E.M. of n rats per group**. Comparison with vehicle (*) is indicated by ** P < 0.01.

Contrarily to B_1_R mRNA levels, the density of B_1_R binding sites was dose-dependently increased at 15 and 50 mg/kg capsaicin (Figure 5). As depicted on autoradiograms, B_1_R binding sites were displayed throughout the grey matter of spinal cord in capsaicin-treated rats and poorly in white matter. Such a distribution is in accordance with B_1_R expression on spinal cord projecting C-fibres, astrocytes and microglia [29].

### Effect of des-Arg^9^-BK on tail-flick latency in capsaicin-treated rats

**Figure 6:**
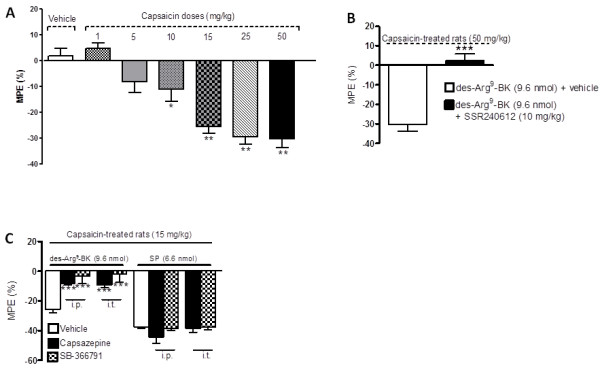
**(A) Effect of des-Arg^9^-BK-induced thermal hyperalgesia in rats treated with capsaicin (1-50 mg/kg, s.c.) or vehicle, 24 h earlier**. (B) Effect of the selective B_1_R antagonist (SSR240612, 10 mg/kg, p.o.) administered 3 h prior to des-Arg^9^-BK in capsaicin-treated rats (50 mg/kg, s.c.). (C) Effect of two TRPV1 antagonists (capsazepine 10 mg/kg, i.p. or 10 μg/site, i.t. and SB-366791 1 mg/kg, i.p. or 30 μg/site, i.t.) given 1h prior to capsaicin on thermal hyperalgesia induced by des-Arg^9^-BK and SP in capsaicin-treated rats (15 mg/kg, s.c.). Shown are the maximal hyperalgesic responses measured at 1 min post-injection of the agonist. Data represent the means ± S.E.M. of 5-7 rats per group. Comparison with the vehicle (*) is indicated by: * P < 0.05; ** P < 0.01; ***P < 0.001.

**Figure 7:**
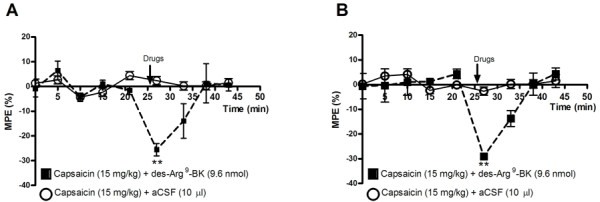
**Time-course effect of des-Arg^9^-BK (9.6 nmol/site, i.t.) on thermal hyperalgesia in the tail-flick test from rats treated 24 h earlier with capsaicin (15 mg/kg, s.c.)**. Shown are the responses in rats housed under constant light (A) or a 12 h-12 h light-dark cycle (B). Data represent the means ± S.E.M. of 4 rats per group. Comparison with the 0 min group (*) is indicated by ** P < 0.01. No statistical significance was seen between the two groups.

**Figure 8:**
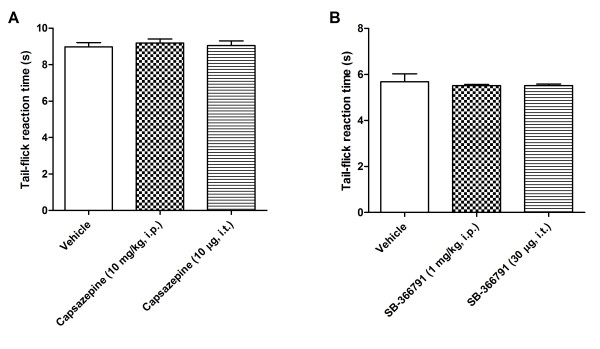
**Tail-flick reaction time of control rats injected with (A) vehicle or capsazepine (10 mg/kg, i.p. or 10 μg/site, i.t.) or (B) vehicle or SB366791 (1 mg/kg, i.p. or 30 μg/site, i.t.)**. Tail-flick reaction time was assessed using either a tungsten lamp (A) or a hot water bath (50°C) as a heat source (B). Data represent the means ± S.E.M. of 4 rats per group.

The functionality of the B_1_R which is up-regulated by capsaicin in the spinal cord was assessed on thermal nociception. Intrathecal injection of des-Arg^9^-BK (9.6 nmol/site) failed to alter nociceptive threshold in control (vehicle) rats or in rats treated with capsaicin at doses of 1 and 5 mg/kg, 24 h earlier (Figure 6A). However, the B_1_R agonist dose-dependently decreased the tail-flick latency in rats treated with doses from 10 to 50 mg/kg capsaicin. The hyperalgesic response to the B_1_R agonist peaked l min post-injection (-30%) and lasted about 5-10 min in rats pre-treated with 15 mg/kg capsaicin (Figure 7). The hyperalgesic response to des-Arg^9^-BK was similar in intensity, onset and duration than that evoked by substance P (6.6 nmol/site, i.t.) [1,31,34]. Des-Arg^9^-BK-induced thermal hyperalgesia in capsaicin-treated rats was completely prevented by the selective B_1_R antagonist SSR240612 (10 mg/kg, p.o.) (Figure 6B) and by both TRPV1-selective antagonists: capsazepine (10 mg/kg, i.p. or 10 μg/site, i.t.) and SB-366791 (1 mg/kg, i.p. or 30 μg/site, i.t.) when injected 1 h prior to capsaicin (Figure 6C). In contrast, similar treatments with TRPV1 antagonists did not affect the hyperalgesic response induced by substance P (6.6 nmol/site, i.t.), showing the specificity of the inhibition (Figure 6C). The TRPV1 antagonists (capsazepine (10 mg/kg, i.p. or 10 μg/site, i.t.) and SB-366791 (1 mg/kg, i.p. or 30 μg/site, i.t.)) had no direct effect on baseline tail-flick reaction time in control rats (Figure 8).

### Housing conditions

Most of the current data were obtained in rats housed under constant light with the intent to prevent the release of endogenous opioid peptides [32] which could interfere with the hyperalgesic response to substance P in the rat tail-flick test [31,34]. To confirm that the interruption of the circadian cycle by constant light did not corrupt our findings, we run a series of experiments with a 12 h-12 h light-dark cycle. These data showed that the time-course pattern of B_1_R agonist-induced thermal hyperalgesia in 15 mg/kg capsaicin-treated rats is similar in rats housed under constant light or under a standard 12 h-12 h light-dark cycle (Figure 7). Moreover, increased B_1_R mRNA levels in the lumbar spinal cord of rats treated with capsaicin (15 and 50 mg/kg, 24 h) under continuous light (Figure 1A) were not significantly different from those measured in rats under the 12 h-12 h light dark-cycle (Figure 1B). Thus, these data confirm that constant light exposure does not interfere with our findings.

### Mechanism underlying des-Arg^9^-BK-induced hyperalgesia

**Figure 9:**
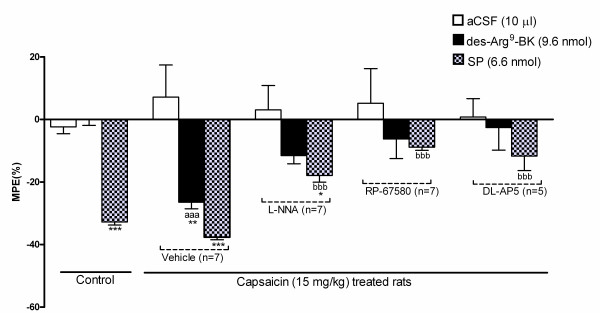
**Effect of several pharmacological treatments on des-Arg^9^-BK- (9.6 nmol/site, i.t.) and SP- (6.6 nmol/site, i.t.) induced thermal hyperalgesia in capsaicin-treated rats (15 mg/kg, s.c., 24 h earlier)**. Shown are the maximal hyperalgesic responses measured 1 min post-injection of the agonist. Rats received intrathecally (10 μg/site, 15 min earlier) either L-NNA, RP-67580, DL-AP5 or vehicle. Control rats were naïve. Data represent the means ± S.E.M. of 5-7 rats in each group. Comparison to aCSF (*), des-Arg^9^-BK in control rats (a) or substance P in control rats (b) is indicated by *P < 0.05, **P < 0.01, *** ^aaa bbb ^P < 0.001.

Whereas control rats treated with SP (6.6 nmol/site, i.t.) showed a significant decrease in tail-flick latency (-34%) when compared with aCSF (-2%), des-Arg^9^-BK (9.6 nmol/site, i.t.) had no effect (0%) (Figure 9). Significant decreases in tail-flick latency were, however, achieved with both des-Arg^9^-BK (-29%) and SP (-41%) in rats treated 24 h earlier with capsaicin (15 mg/kg, s.c.) in comparison with aCSF. A 15-min pre-treatment with either L-NNA (NOS inhibitor; 10 μg/site, i.t.), RP-67580 (NK-1R antagonist; 10 μg/site, i.t.) or DL-AP5 (NMDA-R antagonist; 10 μg/site, i.t.) inhibited significantly the response to both des-Arg^9^-BK and SP. Whereas these inhibitors had no direct effect in control rats (data not shown), they caused antinociceptive effects in capsaicin-treated rats (+51% for L-NNA; +29% for RP-67580 and +9% for DL-AP5). Note that MPE was calculated from baseline values obtained after inhibitor/antagonists administration.

### Microglial localisation of B_1_R in the spinal dorsal horn of capsaicin-treated rats

**Figure 10:**
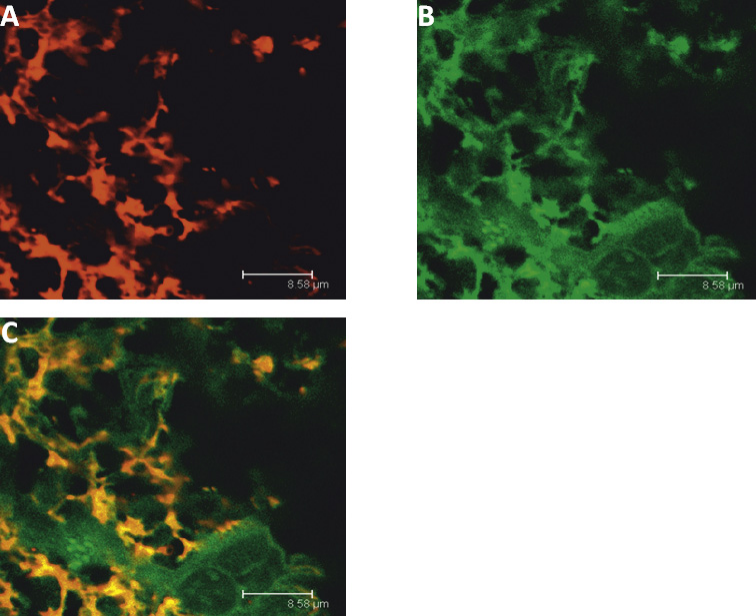
**Confocal microscopy pictures of coronal sections of lumbar spinal cord dorsal horn isolated from 15 mg/kg capsaicin-treated rats**. Microglia were labelled with anti-Iba-1 and are shown in panel A. B_1_R was labelled with N^α^-bodipy-des-Arg^9^-BK and is shown in panel B. Co-localization is shown in yellow in panel C. Images are representative of at least 4 sections/rat from 4 rats/group. Scale bar = 8.58 μmeter. No B_1_R labeling was found in spinal cord of control spinal cord (not shown).

As shown in Figure 10, immunofluorescence to the specific marker of microglia Iba-1 (red, A) and fluorescent labeling with the specific B_1_R agonist [N^α^-bodipy]-des-Arg^9^-BK (green, B) were co-localized (yellow, C) in spinal cord dorsal horn of capsaicin-treated rats (15 mg/kg, s.c., 24 h earlier). Note that 87 ± 4% of B_1_R-positive pixels co-localized with Iba-1-positive pixels, suggesting that B_1_R induced by capsaicin is mostly expressed by lumbar spinal cord microglia.

### Increased superoxide anion production and NF-κB activation in the spinal cord of capsaicin-treated rats

**Figure 11:**
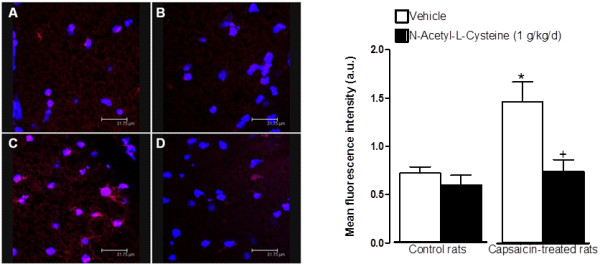
**Confocal microscopy pictures of DHE labelling in spinal cord dorsal horn of 15 mg/kg capsaicin-treated (C, D) and control (A, B) rats, pre-treated for 7 days with the potent antioxidant N-acetyl-L-cysteine (1 g/kg/day) (B, D) or its vehicle (A, C)**. Nuclei were labelled with TO-PRO-3 (blue) and superoxide anion with DHE (red). Scale = 31.75 μm. Data represent the means ± S.E.M. of at least 4 sections/rat from 4 rats/group. Comparison with control + vehicle (*) or capsaicin + vehicle (+) is indicated by: * ^+ ^P < 0.05.

**Figure 12:**
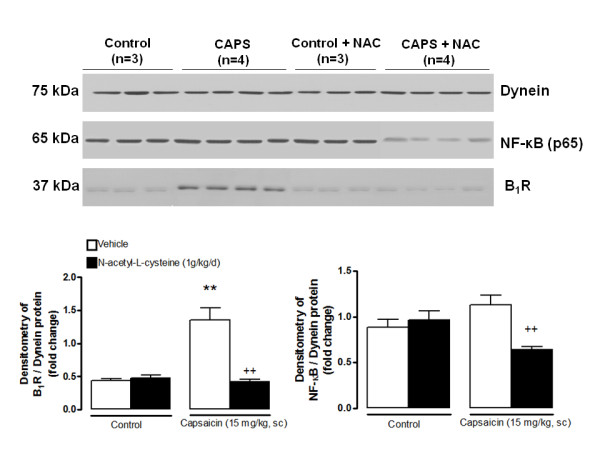
**Control and capsaicin (CAPS, 15 mg/kg, s.c.)-injected rats were pre-treated for 7 days with N-acetyl-L-cysteine (NAC, 1 g/kg/d) or its vehicle**. Protein expression for B_1_R (37 kDa), p65NF-κB (65 kDa) and dynein (70 kDa) was measured on lumbar spinal cord by western blot. Representative immunoblots are shown in the upper panels while densitometric quantifications of protein expression are shown in lower panels. Data represent the means ± S.E.M. of 3-4 rats in each group. Comparison with control + vehicle (*) or capsaicin + vehicle (+) is indicated by ** ++ P < 0.01.

This series of experiments aimed to determine the contribution of oxidative stress to the induction of B_1_R following TRPV1 activation. DHE staining (red staining, Figure 11C) was significantly increased in nuclei (labelled with TO-PRO-3 in blue) in the spinal dorsal horn of rats treated with capsaicin (15 mg/kg, s.c., 24 h earlier) when compared with control spinal cord (Figure 11A). This increase was abolished by a 1-week pre-treatment with NAC (1 g/kg/d) (from 1.46 ± 0.21 to 0.74 ± 0.12 a.u.) (Figure 11D). In contrast, NAC treatment had no significant effect on DHE staining in control spinal cord (Figure 11B). This is in relation with the increased protein expression of B_1_R in the spinal cord of capsaicin-treated rats, which was also prevented by pre-treatment with NAC (Figure 12). These data thus suggest that oxidative stress is involved in the induction of B_1_R by TRPV1 stimulation. To determine the contribution of NF-κB in this process, expression of p65NF-κB was determined. Capsaicin-treatment caused a non-significant increase of p65NF-κB when compared with control spinal cords. However, NAC treatment (1 g/kg/d × 7 days) reduced significantly p65NF-κB expression in the spinal cord of capsaicin-treated rats. NAC had no effect in control spinal cord p65NF-κB expression (Figure 12).

### Changes of IL-1β and TNF-α mRNA levels in the spinal cord of capsaicin-treated rats

**Figure 13:**
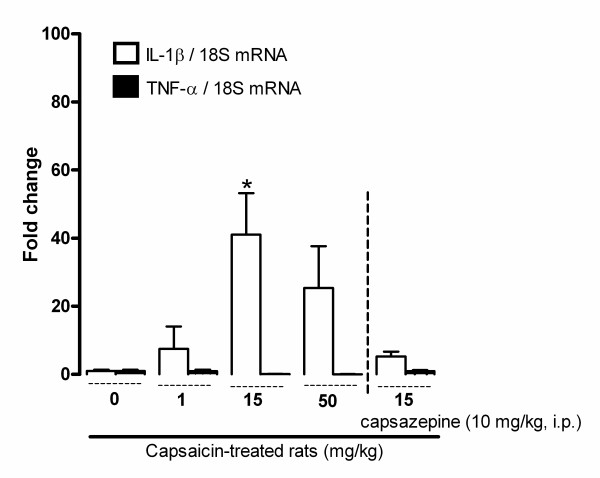
**Changes in IL-1β and TNF-α mRNA levels in lumbar spinal cord of capsaicin-treated rats (0-50 mg/kg, s.c. 24 h earlier)**. Also shown is the effect of the TRPV1 antagonist capsazepine (10 mg/kg, i.p.) administered 1 h prior to capsaicin. Data represent the means ± S.E.M. of 5-7 rats in each group. Comparison with vehicle (0 mg/kg) (*) is indicated by * P < 0.05.

To further determine the mechanism of B_1_R induction by capsaicin, spinal cord mRNA levels of pro-inflammatory cytokines (IL-1β and TNF-α) known to induce B_1_R were assessed using real-time PCR (Figure 13). These data show a significant increase in IL-1β mRNA expression (40-fold) in the spinal cord of capsaicin-treated rats (15 mg/kg, s.c.) when compared with control. This induction was prevented by the selective TRPV1 antagonist capsazepine (10 mg/kg, i.p.) administered 1 h prior to capsaicin. In contrast, TNF-α mRNA levels were unaffected by the three doses of capsaicin. Spinal cord B_1_R and IL-1β mRNA levels following capsaicin challenge were highly correlated (R^2^= 0.996) while mRNA levels of B_1_R and TNF-α (R^2^= 0.562) were not correlated.

## Discussion

The present study provides the first pharmacological evidence that the B_1_R (mRNA and protein levels) can be induced and up-regulated in rat spinal cord after systemic or spinal activation of TRPV1 by capsaicin. The B_1_R induction mechanism involves oxidative stress, pro-inflammatory cytokines and the NF-κB pathway. Activation of TRPV1 on primary sensory afferents and microglial cells can enhance superoxide anion production [26,28], which is known to induce B_1_R expression in various tissues [4,8,15]. Hence, the involvement of oxidative stress in the induction of B_1_R by TRPV1 is consistent with these latter studies, as shown with N-acetyl-L-cysteine treatment that prevented the increased expression of B_1_R and superoxide anion levels in the spinal cord of capsaicin-treated rats. Functionality of the B_1_R was demonstrated in thermonociception. The hyperalgesic response to intrathecally injected B_1_R agonist was ascribed to the intraspinal release of NO and activation of NK-1R and NMDA-R, as previously reported following spinal activation of B_1_R in streptozotocin-diabetic rats [1]. The presence of B_1_R in spinal cord microglia is consistent with the emerging role of microglial B_1_R in pain neuropathy [29,30,49].

### TRPV1 activation and inhibition

In this study, TRPV1 was activated with increasing doses of the lipophilic molecule capsaicin, the active pungent ingredient of hot chilli peppers [50]. Capsaicin's actions are classified into TRPV1-mediated actions (including some side effects) and TRPV1-independent effects. TRPV1-mediated side effects include desensitisation (depletion of SP and CGRP) of C and Aδ neurons following sub-chronic capsaicin treatment, and neuronal apoptosis (calcium neurotoxicity) after chronic treatment with high capsaicin doses [50]. As reviewed by the latter authors, capsaicin TRPV1-independent effects include: alteration of membrane fluidity [51], inhibition of platelet aggregation [52], and toxicity to non-TRPV1 neurons [53]. Hence, to ascertain that the enhancing effect of capsaicin on B_1_R expression is truly mediated by TRPV1 activation, two classes of TRPV1 antagonists (capsazepine and SB-366791) were used [16]. Although high doses of capsazepine can block neuronal voltage-gated calcium channels [54], it has also been shown that capsazepine is 10-100 times more potent for TRPV1 than for its off-target [54]. Secondly, we selected SB-366791 which is known as a more selective and more potent TRPV1 antagonist than capsazepine [42]. Recently, it has been broadly employed as a selective TRPV1 antagonist in pain research [55]. In addition to blocking B_1_R up-regulation induced by capsaicin, capsazepine and SB-366791 reversed selectively des-Arg^9^-BK-induced hyperalgesia without affecting SP-induced hyperalgesia. As additional evidence of specificity, systemic and intrathecal treatments with TRPV1 antagonists (capsazepine and SB-366791) had no direct effect on baseline latency in the tail-flick test. Collectively, these findings strongly suggest that the induction of B_1_R by capsaicin is attributable to TRPV1 stimulation.

### Localisation of B_1_R and site of action for capsaicin

Using a fluorescent B_1_R ligand, B_1_R was mostly found (87% of positive cells) in microglial cells in the lumbar spinal cord dorsal horn of capsaicin-treated rats. Such a cellular localisation on migratory cells can explain the widespread distribution of B_1_R binding sites in all spinal cord laminae. The significant increase of B_1_R mRNA in the spinal cord of capsaicin-treated rats also supports a spinal site rather than a peripheral site (DRG) for B_1_R synthesis.

The induction of B_1_R was largely restricted to lumbar spinal cord segments innervated by peripheral nociceptors in the stimulated region, suggesting a contribution of peripheral TRPV1 to the induction of B_1_R in the spinal cord. The enhanced expression of B_1_R on rostral segments (T4 and C2) of the spinal cord could be due to the expression of B_1_R on migratory microglial cells after stimulation of sensory fibers by capsaicin.

Our data also suggest that induction of B_1_R by capsaicin is mediated by a central mechanism since TRPV1 antagonists administered intrathecally prevented B_1_R mRNA expression and B_1_R-stimulated thermal hyperalgesia. Capsaicin and capsazepine are lipophilic molecules and SB-366791 is likely to pass the blood-brain barrier as well [56]. Therefore, systemically administered capsaicin is thought to enter the spinal cord to stimulate TRPV1 located on central sensory terminals, astrocytes [56] and microglia [56]. Supporting the latter assumption, we showed that 1 μg of capsaicin directly injected into lumbar spinal cord caused a significant increase in B_1_R mRNA, showing that centrally expressed TRPV1 cells can trigger B_1_R expression.

### B_1_R mRNA expression patterns

Contrarily to B_1_R mRNA expression, B_1_R specific binding sites were shown to be maximally increased by 50 mg/kg capsaicin. This is congruent with the faster kinetics of B_1_R mRNA expression after a dose of 50 mg/kg capsaicin in comparison with 15 mg/kg. As B_1_R is hardly internalised or desensitised [6], its presence on the cellular membrane can easily outlast the transient increase of mRNA in response to acute stimulation of TRPV1. Thus, B_1_R mRNA changes do not reflect the expression of B_1_R on the cellular membrane. The actual bell-shaped pattern of B_1_R mRNA expression is not unique and has been observed in other models of acute inflammation induced by exposure to increasing concentrations of tobacco-smoke condensate [48,57].

### Mechanism underlying B_1_R induction

TRPV1-induced oxidative stress is suggested as the primary mechanism by which capsaicin induces B_1_R. This is supported by the suppression of capsaicin-induced increase B_1_R protein expression and NF-kB activation by the 1-week treatment with the antioxidant N-acetyl-L-cysteine. This is in line with the findings of Ma et al. [26], which suggest that TRPV1 activation by capsaicin increases reactive oxygen species production in mouse dorsal root ganglion neurons. This can occur through increased cytosolic calcium influx and activation of NADPH oxidase [27]. N-acetyl-L-cysteine also prevented the increased superoxide anion production in the spinal cord of capsaicin-treated rats. It is noteworthy that N-acetyl-L-cysteine or a prolonged treatment with another antioxidant (alpha-lipoic acid) has been shown to prevent rat spinal cord B_1_R induction in models of diabetes [8,15] and hypertension [58]. In a model of rat knee joint-induced arthritis, peripheral TRPV1 and centrally increased oxidative stress can enhance pro-inflammatory cytokines production [59], suggesting that these inflammatory molecules can also contribute to the effect of capsaicin on B_1_R expression. Indeed, stimulation of TRPV1 causes the release of various pro-inflammatory cytokines, including interleukin-6 (IL-6) and IL-8 from human bronchial epithelial cells [60], IL-1ß and transforming growth factor-ß_2 _from human ORS keratinocytes [61], IL-2, IL-4 and interferon-gamma from cultured murine Peyer's patch cells [62], or IL-6 release and NF-κB activation in dorsal horn TRPV1-expressing microglia [63]. Those pro-inflammatory cytokines could activate NF-κB translocation to the nucleus, thereby increasing B_1_R expression [1,64,65]. One can suggest that cytokines may represent key mediators in the induction of B_1_R following TRPV1 activation. As indirect evidence, we found that IL-1β mRNA levels are enhanced and highly correlated with B_1_R mRNA levels following TRPV1 stimulation. In contrast, TNF-α is unlikely to be involved in B_1_R expression as its mRNA level was unaffected by capsaicin administration. This is in agreement with data showing no correlation between B_1_R induction and TNF-α expression in tobacco smoke-induced lung inflammation [48].

### B_1_R activation led to thermal hyperalgesia

TRPV1 activation by capsaicin induced functional B_1_R in the rat spinal cord. This is highlighted by the hyperalgesia induced by intrathecal administration of B_1_R selective agonist (des-Arg^9^-BK) in capsaicin-treated rats. This response was inhibited by SSR240612, a highly selective and orally active B_1_R antagonist [38]. In contrast, des-Arg^9^-BK had no effect on the nociceptive threshold in control rats which is congruent with its weak constitutive expression. The spinal cord activation of tachykinin NK-1 receptor with SP causes a hyperalgesic response in the tail-flick test which is associated with glutamate release and NO production. Indeed, blockade of NOS or NMDA receptor prevents thermal hyperalgesia induced by endogenous release of SP following noxious cutaneous stimulation or by intrathecal administration of SP [66-68]. The hyperalgesic response induced by SP has the same time-course and amplitude as that induced by des-Arg^9^-BK. Also, our pharmacological analysis shows that the hyperalgesic response to B_1_R agonist is likely mediated by endogenous release of NO and activation of NK-1R and NMDA-R. These spinal pro-nociceptive mediators appear to be tonically active in capsaicin-treated rats as their inhibition increased the nociceptive threshold. Hypoalgesia has also been reported with inhibition of NOS with L-NNA in streptozotocin-diabetic rats [1] or with NMDA receptor blockade [69]. Since the hyperalgesic response to SP in capsaicin-treated rats was not significantly altered, the hyperalgesic response to des-Arg^9^-BK is unlikely to be due to an upregulation of NK-1 receptors or increased SP response in the spinal cord of capsaicin-treated rats. The TRPV1 antagonists capsazepine and SB-366791 prevented the hyperalgesia induced by des-Arg^9^-BK but not that evoked by SP, suggesting that this inhibition is highly specific.

## Conclusion

This study provides the first evidence that B_1_R can be induced and up-regulated in microglia of spinal cord dorsal horn following activation of TRPV1 by capsaicin. This up-regulation is correlated with increased expression of IL-1β and involves ROS generation and the redox-regulated NF-κB pathway. The newly synthesized B_1_R is functional as its activation with an agonist caused thermal hyperalgesia which we attribute to the intraspinal release of NO and activation of NK-1R and NMDA-R. These findings support the possibility that microglial B_1_R contributes to the effect of TRPV1 in inflammatory pain.

## List of abbreviations

ANOVA: Analysis of variance; anti-Iba-1: anti-Ionized calcium binding adapter molecule 1; aCSF: artificial cerebrospinal fluid; a.u.: arbitrary unit; BK: bradykinin; BSA: bovine serum albumin; DL-AP5: D,L-2-amino-5-phosphonovaleric acid; DNA: Deoxyribonucleic acid; des-Arg^9^-BK: des-Arg^9^-bradykinin; DHE: dihydroethidium; DMSO: dimethyl sulfoxide; DRG: dorsal root ganglion; EtBr: ethidium bromide; GPCR: G-protein-coupled receptor; HPP-desArg^10^-Hoe140: 3-(4 hydroxyphenyl) propionyl-desArg^9^-D-Arg^0^[Hyp^3^, Thi^5^, D-Tic^7^, Oic^8^]bradykinin; IASP: International Association for the Study of Pain; IL-1β: interleukin-1 beta; i.t.: intrathecal; B_1_R: kinin B_1 _receptor; B_2_R: kinin B_2 _receptor; L-NNA: L-NG-Nitroarginine; MPE: maximum possible effect; mRNA: messenger RNA; NAC: N^α^-4,4,-difluoro-5,7-dimethyl-4-bora-3a,4a-diaza-s-indacene-3-propionic acid succinimidyl ester-des(Arg^9^)-Bradykinin [N^α^-bodipy]-des-Arg^9^-BK; N-acetyl-L-cysteine; NADPH: nicotinamide adenine dinucleotide phosphate-oxidase; NO: nitric oxide; NOS: nitric oxide synthase; NK-1: neurokinin-1; NMDA: N-Methyl-D-aspartic Acid; PCR: polymerase chain reaction; PBS: phosphate buffered saline; qRT-PCR: quantitative real-time PCR; R-715: AcLys[D-βNal^7^,Ile^8^]des-Arg^9^-BK; ROS: reactive oxygen species; SB-366791: N-(3-methoxyphenyl)-4-chlorocinnamide; RP-67580: Imiro-1 (methoxy-2 phenyl)-2ethyl]-2 diphenyl-7.7 perhydroisoindolone-4-(3aR, 7aR); SSR240612: [(2R)-2-[((3R)-3-(1,3-benzodioxol-5-yl)-3-[[(6-methoxy-2-naphthyl)sulfonyl]amino]propanoyl)amino]-3-(4-[[2R,6S)-2,6-dimethylpiperidinyl]methyl]phenyl)-N-isopropyl-N-methylpropanamide,fumarate]; SP: substance P; NF-kappa B: transcriptional nuclear factor kappa B; TRPV1: transient receptor potential vanilloid 1; TNF-α: tumor necrosis factor alpha.

## Competing interests

The authors declare that they have no competing interests.

## Authors' contributions

ST and JPD performed the experiments. ST designed the study, analyzed the data and wrote the manuscript. KL carried out western blot analysis. MRB gave us access to his real-time PCR apparatus. MMC helped in study design and data analysis. PG conceived and synthesized the B_1_R fluorescent agonist. RC conceived experiments, supervised the work and wrote the final version of the manuscript. All authors have read and approved the final version of the manuscript.
